# Sulforaphane alleviates hepatocyte pyroptosis via activating Nrf2-HO-1 signaling during septic acute liver injury

**DOI:** 10.3389/fphar.2025.1690067

**Published:** 2025-10-16

**Authors:** Shiying Xie, Min Liu, Lei Chen, Yincai Xie, Li Liu, Weina Chen, Huanwen Huang

**Affiliations:** ^1^ Department of Pharmacy, The Fifth Affiliated Hospital (Zhuhai) of Zunyi Medical University, Zhuhai, Guangdong, China; ^2^ Department of Urology, 900th Hospital of Joint Logistic Support Force, Fuzhou, Fujian, China

**Keywords:** acute liver injury, lipopolysaccharide, sulforaphane, pyroptosis, Nrf2

## Abstract

**Objective:**

Acute liver injury (ALI) caused by sepsis is a fatal disease with a high mortality rate and poor prognosis. Sulforaphane (SFN) is a natural isothiocyanate that has robust antioxidant and anti-inflammatory properties. The aim of this study was to identify the pharmacological effects and therapeutic mechanisms of SFN in lipopolysaccharide (LPS)-induced ALI.

**Methods:**

The role of SFN in ALI was investigated using a mouse model of LPS-induced ALI. Briefly, eighteen mice were divided into three groups: control, LPS, and LPS + SFN, which were intraperitoneally injected for 2 days before LPS treatment. 24 h after the LPS injection, blood and liver tissues were collected for further analysis.

**Results:**

The hematoxylin and eosin (HE) staining showed a lot of visible necrosis areas, inflammatory cell infiltration, and congestion in liver. Meanwhile, Ly6G and F4/80 staining showed increased infiltration of neutrophils and macrophages in liver, these results indicated that LPS induced sever ALI. As inflammatory response plays a vital role in the pathogenesis of LPS-induced ALI, we detected the occurrence of pyroptosis in liver by ribonucleic acid (RNA) sequencing. The results showed that pyroptosis was significantly promoted by LPS, as indicated by gene ontology (GO) and kyoto encyclopedia of genes and genomes (KEGG) pathway enrichment analyses, revealing the activation of pyroptosis, interleukin (IL)-1 production, IL-18 production, and inflammatory signaling pathways. Then, we explored the effect of SFN on LPS-induced ALI. The results showed that SFN obviously reduced LPS-induced plasma alanine aminotransferase and aspartate aminotransferase level, pathological injuries and TdT-mediated dUTP nick-end labeling (TUNEL) positive cells, indicating protective effect of SFN on ALI. Furthermore, SFN also showed robust effect on LPS-induced inflammatory response in liver, as reflected by suppressing the infiltration of neutrophils and macrophages, and downregulating mRNA levels of C-X-C motif chemokine ligand 9, IL-6, monocyte chemoattractant protein-1, and tumor necrosis factor-α in liver of LPS treated mice. Furthermore, SFN blocked hepatocyte pyroptosis, and suppressed plasma IL-1β and IL-18 levels of LPS treated mice. Mechanistically, SFN selectively activated nuclear factor erythroid 2-related factor 2 (Nrf2)/heme oxygenase-1 (HO-1) signaling to mediate pyroptotic cell death. SFN also reversed the inhibited superoxide dismutase activity and induced malondialdehyde content in liver of LPS exposed mice.

**Conclusion:**

SFN ameliorated liver injury and inflammation during LPS-induced ALI by suppressing hepatocyte pyroptosis via the activation of Nrf2/HO-1 signaling. This study provides new evidence for the potential treatment of ALI with SFN.

## 1 Introduction

Sepsis, characterized by life-threatening organ dysfunction, is a leading cause of death in the intensive care units (Cecconi et al., 2018). Multiple organs are affected during sepsis, and the liver is one of the most commonly affected organs by pro-inflammatory factors, which leads to poor clinical outcomes (Lelubre and Vincent, 2018; Zhang et al., 2022). However, therapeutic strategies to efficiently attenuate sepsis-induced acute liver injury (ALI) are still lacking, implying that a more comprehensive understanding of sepsis-induced liver injury pathogenesis is required.

Pyroptosis, a gasdermin-mediated programmed cell death, is triggered by inflammasome-regulated autoproteolysis of caspases and is characterized by cell swelling, perforation, lysis, and release of cell contents (Yu et al., 2021). Activated inflammasomes initiate cleavage of caspases, which then regulate the maturation of gasdermin, interleukin (IL)-1β and IL-18 precursors (Yu et al., 2021; Shi et al., 2017). Subsequently, the cell membrane is disrupted by the pores formed by the N-terminus of gasdermins, cytokines such as IL-1β and IL-18, and molecules such as high mobility group box 1 and adenosine triphosphate, are released, triggering an innate immune response (Yu et al., 2021; Shi et al., 2017). During the process of pyroptosis, Gasdermin D (GSDMD) plays a central role as the key effector molecule. GSDMD contains a 31 kDa N-terminus (GSDMD-N) and a 22 kDa C-terminus (GSDMD-C) which is connected by a peptide linker (Shi et al., 2017). Once activated, the linker is cleaved to separate GSDMD-N from GSDMD-C. GSDMD-N forms a transmembrane pore that releases cytokines, and also disturbs the regulation of ions and water, eventually resulting in pyroptosis (Shi et al., 2017). Although pyroptosis is essential for pathogen defence, excessive pyroptosis may contribute to chronic inflammation and inflammatory diseases (Wei et al., 2023; Chen X. et al., 2023; Xie et al., 2024a). Recent studies have reported the prominent role of pyroptosis in ALI, including blocking hepatocyte pyroptosis, which significantly relieves ischemia-induced ALI (Kadono et al., 2022; Chen R. X. et al., 2023).

Oxidative stress is the leading cause of pyroptotic cell death (Miao et al., 2023; Wang et al., 2024). Nrf2 (nuclear factor-erythropoietin 2-related factor 2), a basic leucine zipper redox-sensitive transcription factor, is a major regulator of cytoprotective genes with antioxidant and anti-inflammatory properties (Cuadrado et al., 2019). Under static conditions, Nrf2 interacts with Kelch-like ECH-associated protein 1 (Keap1) and is inactive in the cytoplasm (Cuadrado et al., 2019). Upon exposure to oxidative stress, Nrf2 is released from Keap1 and translocates to the nucleus to activate its downstream genes to defend against oxidative stress and inflammation (O’Rourke et al., 2024; Wei et al., 2021). Among these genes, heme oxygenase-1 (HO-1) emerges as a vital target protein of Nrf2 and functions as a rate-limiting enzyme that degrades heme into carbon monoxide, free iron and biliverdin (Wang et al., 2023). Studies have shown that the activation of Nrf2 and induction of HO-1 can block hepatocyte pyroptosis in nonalcoholic fatty liver disease, indicating that interventions targeting Nrf2 and HO-1 may alleviate hepatocyte pyroptosis (Liu et al., 2024). Furthermore, Nrf2 is a critical target in alleviating ALI, previous studies have demonstrated that Nrf2 activators, such as artemisitene and dimethyl fumarate, could obviously ameliorate septic ALI (Giustina et al., 2018; Zhao et al., 2023). While Nrf2 knockout significantly aggravated septic ALI (Xie et al., 2023). However, identification of molecular targets that effectively inhibit pyroptosis and block lipopolysaccharide (LPS)-induced ALI pathogenesis remains elusive.

Sulforaphane (SFN), a natural isothiocyanate compound found in cruciferous vegetables with robust antioxidant and anti-inflammatory properties, is the most potent natural inducer of Nrf2 (Ma et al., 2023; Russo et al., 2018). The potent therapeutic effects of SFN on diseases and its low cytotoxicity have been extensively proved (Ma et al., 2023; Russo et al., 2018). Especially, compared to synthesized Nrf2 agonists, SFN activates Nfrf2 via inhibiting Keap1 which is indirect and saturable, but not binding or modifying the specific cysteine residue on Keap1 which may cause constant hyperactivation of Nrf2 (Cuadrado et al., 2019; Kensler et al., 2007). SFN has been shown to have promising therapeutic effects in a variety of acute and chronic inflammatory diseases, for example, SFN alleviated exhaustive exercise-induced skeletal muscle inflammation via Nrf2/HO-1 signaling (Ma et al., 2023; Ruhee et al., 2025). However, the functional role and potential regulatory mechanism of SFN in LPS-induced ALI remains to be investigated.

While the protective role of the Nrf2/HO-1 pathway in LPS-induced ALI and the detrimental role of pyroptosis have been individually established, the direct link between them remains further exploration. Our findings firstly demonstrate that SFN alleviates LPS-induced ALI predominantly by suppressing hepatocyte pyroptosis via activation of the Nrf2/HO-1 pathway. We applied SFN to LPS-induced ALI focusing on pyroptosis and also provided a theoretical framework for treating LPS-induced ALI with SFN.

## 2 Materials and methods

### 2.1 Mice and treatments

Male C57BL/6J mice (n = 18, 8 weeks old) were purchased from Gempharmatech (Nanjing, China) and maintained in a temperature-controlled room under a 12:12-h light–dark cycle for 2 weeks, with free access to tap water and standard mouse chow. Male C57BL/6J mice were chosen due to their well-characterized response to septic acute liver injury and to avoid potential variance introduced by the female estrous cycle (Shendge et al., 2024; Liu et al., 2020; Clayton and Collins, 2014; Martino et al., 2025). A group size of n = 6 was chosen based on power analysis to detect a significant effect size observed in prior similar studies (Hu et al., 2025; Li et al., 2025). Intraperitoneal injection of LPS was used to induce ALI, as it is a well-established and reproducible model that recapitulates the acute hyper-inflammatory phase of sepsis-induced acute liver injury (Shendge et al., 2024; Liu et al., 2020). And then, the mice were randomly divided into three groups (*n* = 6 per group): Control, LPS (cat# L2630, Sigma, Saint Louis, United States) and LPS + SFN (cat# HY-13755, MCE, Shanghai, China). Mice were intraperitoneally administered SFN (10 mg/kg/day) for 2 days before LPS (10 mg/kg) treatment. All animals were sacrificed 24 h after LPS injection, and samples were collected. This animal study was approved by the Ethics Committee for Animal Experimentation at the Fifth Affiliated Hospital of Zunyi Medical University, and all animal procedures were performed in accordance with the Guide for Care and Use of Laboratory Animals. This study was approved by the Ethics Committee of the Zunyi Medical University (protocol code: ZMU21-2407-014).

### 2.2 Plasma ALT and AST measurement

Plasma alanine aminotransferase (ALT) and aspartate aminotransferase (AST) levels were measured using commercial diagnostic kits (cat# C009-2-1 and cat# C010-2-1, Nanjing Jiancheng Bioengineering Institute) according to the manufacturer’s instructions. The absorbance was measured using a microplate reader (EnVision, PerkinElmer, Waltham, MA, United States).

### 2.3 Western blotting

Western blotting was performed as previously described (Xie et al., 2024b). Briefly, liver tissues were lysed and sonicated in RIPA lysis buffer (cat# BL504A, Biosharp, Hefei, China) containing proteinase and phosphatase inhibitors on ice. Protein concentration was quantified using BCA reagent (cat# P0010, Beyotime, Shanghai, China). Equivalent amounts of protein samples were used for the Western blot analysis. The Primary antibodies used were anti-GSDMD-N, 1:1,000 (cat# ab215203, Abcam, Cambridge, United Kingdom), anti- Nrf2, 1:1,000 (cat# sc-722, Santa Cruz Biotechnology, Dallas, United States), and anti-HO-1, 1:1,000 (cat# 10701-1-AP, Proteintech, Wuhan, China).

### 2.4 MDA and SOD enzyme activity measurement

The malondialdehyde (MDA) concentration in liver tissues was assessed using an assay kit purchased from Nanjing Jiancheng Bioengineering Institute (cat# A003-2-2, Nanjing, China) according to the manufacturer’s instructions. Superoxide dismutase (SOD) enzymatic activity in liver tissues was assayed using a SOD Assay Kit with WST-8 (cat# S0103, Beyotime Company, Shanghai, China) following the manufacturer’s instructions. The absorbance was measured using a microplate reader (EnVision, PerkinElmer, Waltham, MA, United States).

### 2.5 TUNEL staining

To detect TdT-mediated dUTP nick-end labeling (TUNEL)-positive cells in the liver sections, the TUNEL BrightGreen kit was used according to the manufacturer’s instructions (cat# A112-03, Vazyme, Nanjing, China). Green fluorescence-labeled cells were captured using a fluorescence microscope (Leica, Wetzlar, Germany). The immunofluorescence slides were viewed by a blinded investigator using a microscope at 40x magnification (Leica, Wetzlar, Germany). Five unique and representative fields from each sample were selected for further analysis. The average results from each sample were applied for further comparison of different treatments to avoid selection bias. No overlap of chosen fields was allowed during imaging, and those areas with deformations or holes were also excluded during imaging.

### 2.6 Quantitative real-time-PCR

Total RNA was purified from the cells using TRIzol reagent (cat# 15596026CN, Invitrogen, Carlsbad, CA, United States) according to the manufacturer’s instructions. Reverse transcription was performed using the PrimeScript™ RT Master Mix (cat # RR036A, Takara, Kyoto, Japan). Quantitative real-time PCR was performed using SYBR Green qPCR Master Mix (cat. # HY-K0501A; MCE, Shanghai, China) on a StepOnePlus Real-Time PCR System (Applied Biosystems, Waltham, MA, United States). The expression levels of target genes were normalized to β-actin. The primers used in this paper were obtained from OriGene.

### 2.7 RNA-seq analysis

Total RNA was extracted from the liver using TRIzol reagent (cat# 15596026CN, Invitrogen, Carlsbad, United States) and quantified using a NanoDrop Microvolume Spectrophotometer (Thermo Fisher Scientific, Waltham, United States). 1 μg of total RNA was used to construct RNA-sequencing (RNA-seq) libraries. RNA-seq was performed using the MGISEQ2000 platform (BGI Genomics), which generated clean reads. Each RNA-seq read was mapped to the mouse genome using Cutadapt (v1.9.1) with default values for the parameters. The mappable reads were assembled using HISAT2 (v2.2.1) with the default parameters. FPKM (fragments per kilobase per million mapped reads) values were called using Cufflinks.

### 2.8 Bioinformatics analysis

Differential gene expression analysis of RNA-seq data was performed using the DESeq2 package (version 1.48.1). Gene set enrichment analysis (GSEA) was done with the ClusterProfiler package (version 4.14.6) and visualized using the ggplot2 package (version 3.5.2) and enrichplot package (version 1.26.6). Pyroptosis-related gene signatures were obtained from the Molecular Signatures Database (https://www.gsea-msigdb.org/gsea/msigdb/).

### 2.9 Histological analysis

The liver tissues were fixed overnight in 4% paraformaldehyde, dehydrated in a graded series of ethanol solutions, and embedded in paraffin. For histopathological analysis, 3 μm sections were stained with hematoxylin and eosin (HE) using standard procedures. Bright-field images were randomly captured by a blinded investigator using a microscope at 20x magnification (Leica, Wetzlar, Germany).

### 2.10 Immunohistochemistry

Immunohistochemistry (IHC) was performed as previously described (Xie et al., 2024a). Briefly, paraffin-embedded liver sections were prepared in a routine procedure, and then incubated with primary antibodies at 4 °C overnight, followed by incubation with biotinylated secondary antibodies for 1 h at room temperature, followed by incubation with streptavidin-biotin complex for another 1 h (Cat#BA1088, Boster, Wuhan, China). The primary antibody dilutions used were as follows, F4/80, 1:1,000 (Cat#70076S, Cell Signaling Technology, Boston, MA, United States) and Ly6G, 1:500 (Cat#ab238132, Abcam, Cambridge, United Kingdom). DAB was used to visualize antibody labeling, and hematoxylin was used to counterstain the slices. Bright-field images were randomly captured by a blinded investigator using a microscope at 40x magnification (Leica, Wetzlar, Germany). Five unique and representative fields from each sample were selected for further analysis. The average results from each sample were applied for further comparison of different treatments to avoid selection bias. No overlap of chosen fields was allowed during imaging, and those areas with deformations or holes were also excluded during imaging.

### 2.11 IL-1β and IL-18 ELISA

The concentrations of plasma interleukin (IL)-1β or IL-18 in mice were determined using an IL-1β mouse ELISA kit (Cat# SEA563Mu, Cloud-Clone Corporation, Wuhan, China) or IL-18 mouse ELISA kit (Cat# SEA064Mu, Cloud-Clone Corporation, Wuhan, China) according to the manufacturer’s protocols. The absorbance was measured using a microplate reader (EnVision, PerkinElmer, Waltham, MA, United States).

### 2.12 Statistical analysis

All quantitative data are presented as mean ± SEM. One-way ANOVA was used for comparisons including more than two groups, and Tukey’s *post hoc* test was used to estimate the significance between two groups once significant differences were found. Differences were considered statistically significant at P < 0.05. All statistical analyses were performed using the GraphPad Prism software V8.4.3. Data were first tested for normality using the Shapiro-Wilk test and for homogeneity of variances using Brown-Forsythe test. After confirming that the data met the assumptions of normality and equal variance, one-way analysis of variance (ANOVA) was conducted to determine overall significance, followed by Tukey’s *post hoc* test for multiple comparisons. A p-value of less than 0.05 was considered statistically significant.

## 3 Results

### 3.1 Liver injury and pyroptosis is activated by LPS

**FIGURE 1:**
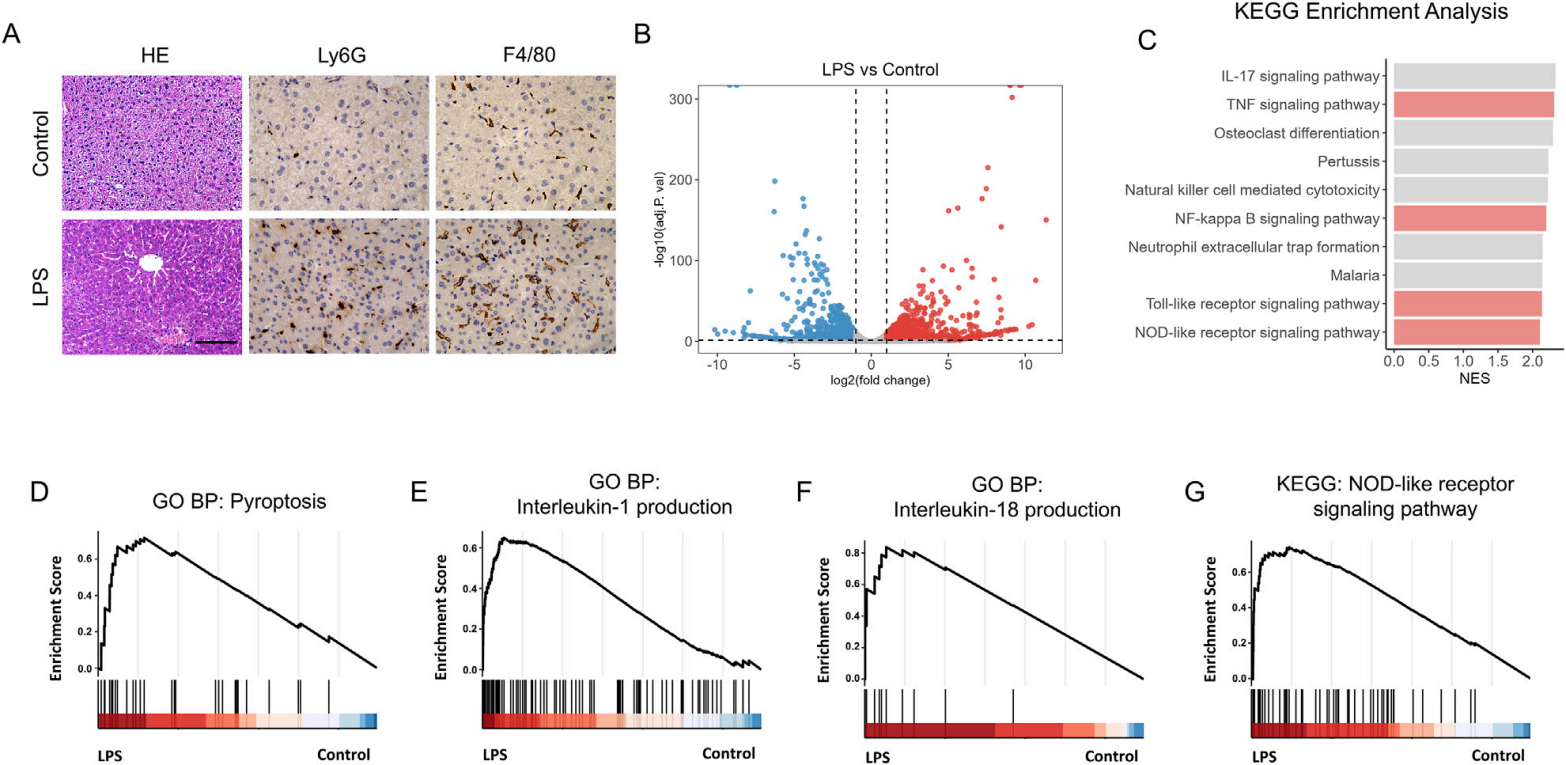
Liver injury and pyroptosis is activated by LPS. **(A)** Hematoxylin–Eosin (HE) staining of control and LPS-treated mice. Scale bar, 20 μm, 40x magnification. n = 6 per group. 5 unique and representative fields from each sample were selected for further analysis. **(B)** Volcano plot showing the differentially expressed genes in liver of control and LPS treated mice. **(C)** Kyoto Encyclopedia of Genes and Genomes (KEGG) enrichment analysis reveals that several biological processes were enriched. **(D–G)** GSEA analysis of pyroptosis **(D)**, Interleukin-1 production **(E)**, Interleukin-18 production **(F)** and NOD-like receptor signaling pathway **(G)** in liver of control (n = 4) and LPS treated mice (n = 3).

To set up the animal model of septic acute liver injury (ALI), lipopolysaccharide (LPS) was used. And the hematoxylin and eosin (HE) analysis showed that liver was significantly injured by LPS treatment (Figure 1A). Meanwhile, neutrophils and macrophages obviously infiltrated the liver in LPS treated mice (Figure 1A), indicating the aggravated inflammatory response. Pyroptosis is a type of programmed cell death closely associated with inflammatory responses. Ribonucleic acid (RNA) sequencing was performed to explore the correlation between pyroptosis and LPS-induced ALI. Differential gene expression analysis is shown as a volcano plot (Figure 1B). Kyoto Encyclopedia of Genes and Genomes (KEGG) enrichment analysis revealed that the TNF, NF-kappa B, Toll-like receptor, and NOD-like receptor signaling pathways were significantly activated (Figure 1C). Furthermore, Gene Ontology (GO) enrichment analysis showed that the genes upregulated under LPS stimulation were associated with pyroptosis, interleukin (IL)-1 production, IL-18 production, and the NOD-like receptor signaling pathway (Figures 1D–G). These results suggest the activation of hepatocyte pyroptosis in LPS-exposed mice.

### 3.2 SFN protects against LPS-induced acute liver injury

**FIGURE 2:**
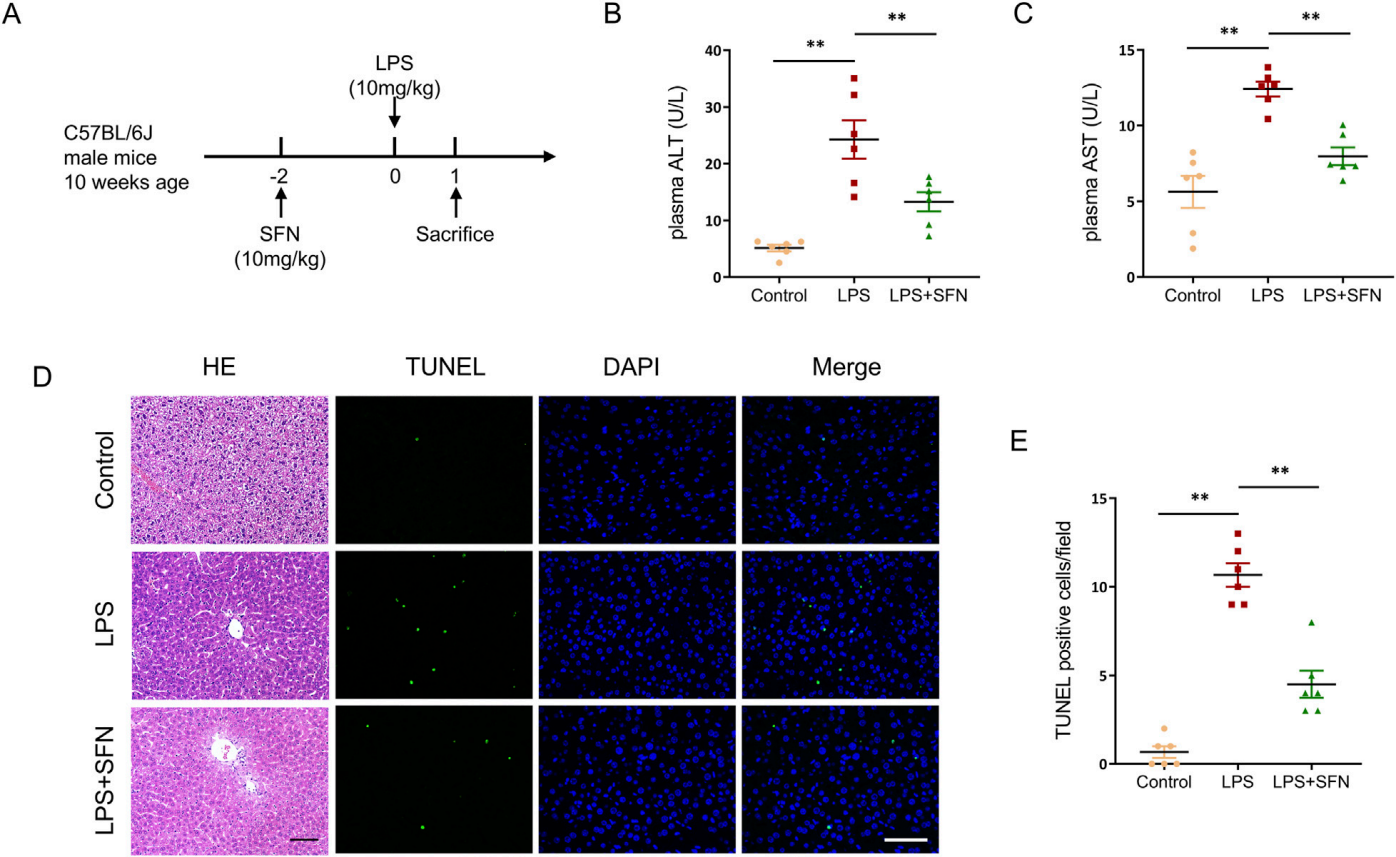
SFN protects against LPS-induced acute liver injury. **(A)** Schematic diagram of the experimental design. **(B,C)** Plasma ALT and AST detection of control and LPS-treated mice with or without SFN pretreatment. **(D,E)** Representative images of HE staining and TUNEL staining in liver tissues from different groups **(D)**, and quantitative analysis of TUNEL positive cells **(E)**. Scale bar, 10 μm, 20x magnification. Five unique and representative fields from each sample were selected for further analysis. Data are presented as mean ± SEM of biologically independent samples. *P < 0.05, **P < 0.01. n = 6 per group. One-way ANOVA was used to analyze the data among multiple groups, followed by Tukey’s *post hoc* test.

To investigate the effect of sulforaphane (SFN) on LPS-induced ALI, an LPS-induced ALI mouse model pre-treated with SFN was used. Liver tissues and blood samples were collected from mice 24 h after LPS injection (Figure 2A). Biochemical analyses showed that SFN decreased LPS-induced plasma levels of alanine aminotransferase (ALT) and aspartate aminotransferase (AST) (Figures 2B,C). Meanwhile, SFN reduced pathological injuries in ALI mice, which exhibited obvious cell ballooning, degeneration, and necrosis of hepatocytes (Figure 2D). Furthermore, TdT-mediated dUTP nick-end labeling (TUNEL) staining showed that LPS exacerbated hepatocyte cell death, which was alleviated by SFN treatment (Figures 2D,E). These results suggested that SFN attenuated LPS-induced acute hepatotoxicity.

### 3.3 SFN alleviated LPS induced inflammatory response in liver

**FIGURE 3:**
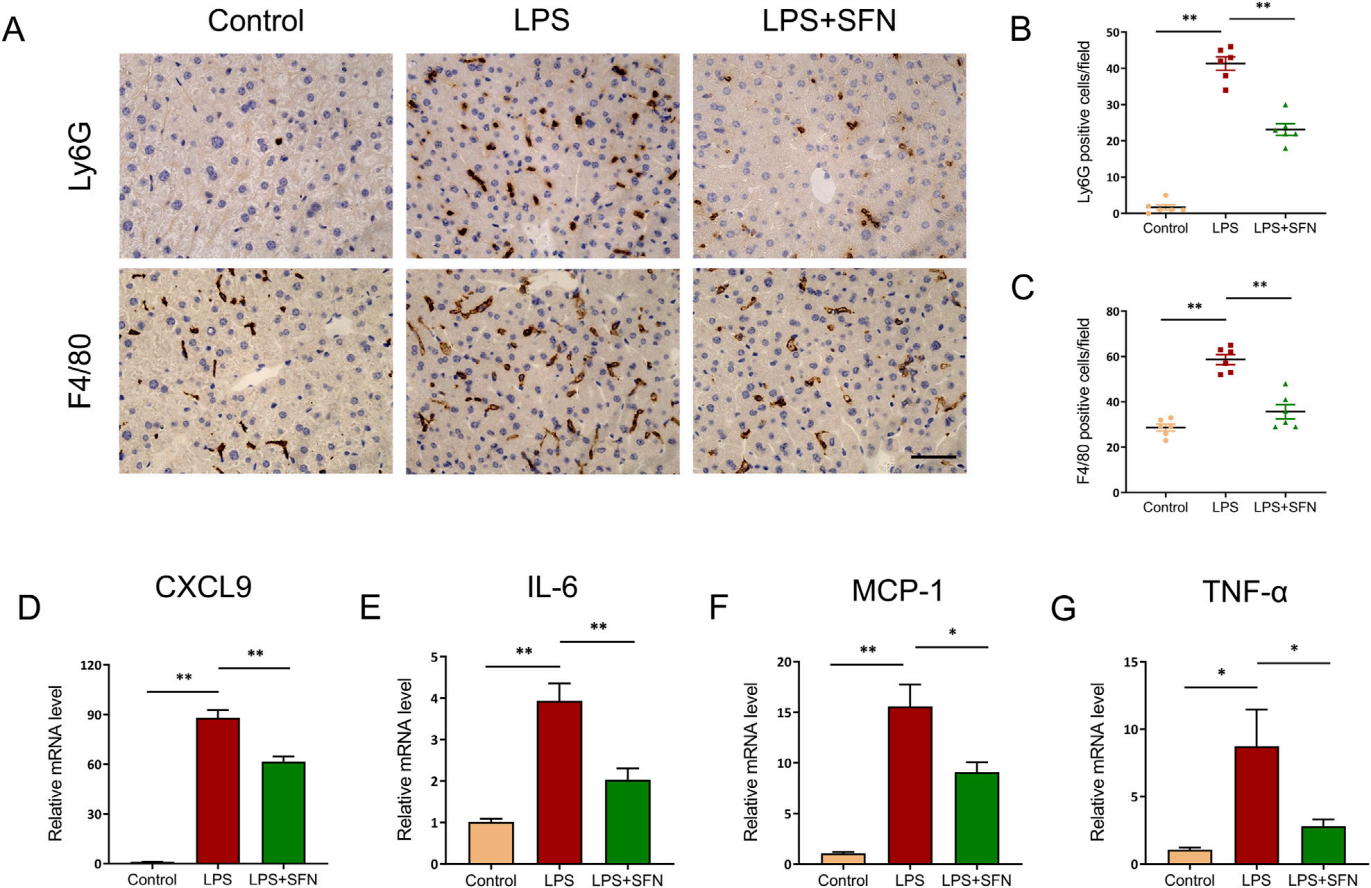
SFN alleviated LPS induced inflammatory response in liver. **(A–C)** Representative images of Ly6G and F4/80 staining of control and LPS-treated mice with or without SFN pretreatment **(A)**, and quantitative analysis **(B,C)**. Scale bar, 10 μm, 20x magnification. Five unique and representative fields from each sample were selected for further analysis. **(D–G)** mRNA expression of CXCL9, IL-6, MCP-1 and TNF-α in liver tissues from different groups. Data are presented as mean ± SEM of biologically independent samples. n = 6 per group. *P < 0.05, **P < 0.01. One-way ANOVA was used to analyze the data among multiple groups, followed by Tukey’s *post hoc* test.

Next, we examined the effect of SFN on hepatic inflammatory response in LPS-treated mice. Immunohistochemical staining showed that SFN suppressed the hepatic inflammatory response in LPS-treated mice, as reflected by the markedly reduced infiltration of Ly6G^+^ neutrophils and F4/80^+^ macrophages (Figures 3A–C). Furthermore, compared with the LPS group, SFN significantly inhibited the mRNA levels of C-X-C motif chemokine ligand 9 (CXCL9), IL-6, monocyte chemoattractant protein-1 (MCP-1), and tumor necrosis factor-α (TNF-α) (Figures 3D–G). Collectively, these data indicate that SFN relieves hepatic inflammation in mice with sepsis.

### 3.4 SFN inhibited hepatocyte pyroptosis in ALI mice

**FIGURE 4:**
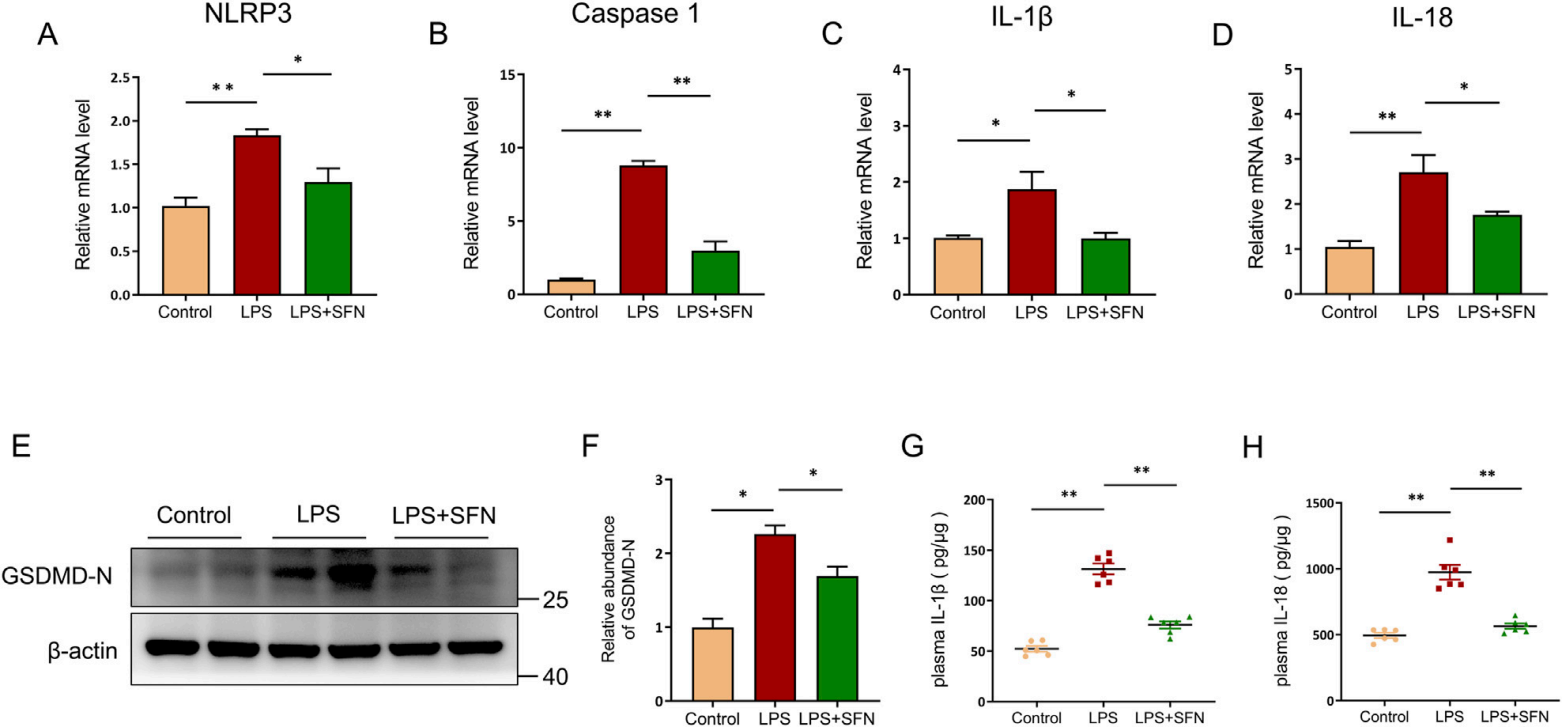
SFN inhibited hepatocyte pyroptosis in ALI mice. **(A–D)** mRNA expression of NLRP3, Caspase 1, IL-1β and IL-18 in liver tissues from control and LPS-treated mice with or without SFN pretreatment. **(E,F)** Western blot analysis of GSDMD-N in different groups of mice **(E)**, and quantitative analysis **(F)**. **(G,H)** Plasma IL-1β and IL-18 detection in different groups. Data are presented as mean ± SEM of biologically independent samples. n = 6 per group. *P < 0.05, **P < 0.01. One-way ANOVA was used to analyze the data among multiple groups, followed by Tukey’s *post hoc* test.

Next, we explored the effects of SFN on pyroptosis in hepatocytes. RT-qPCR analysis revealed that SFN reduced the expression of pyroptosis-associated genes, including NLRP3, Caspase 1, IL-1β, and IL-18 (Figures 4A–D). A similar trend was observed in GSDMD-N abundance (Figures 4E,F). Furthermore, LPS upregulated the plasma levels of IL-1β and IL-18, which were suppressed by SFN treatment (Figures 4G,H). These results showed that SFN blocked hepatocyte pyroptosis in LPS-induced ALI.

### 3.5 SFN activated Nrf2/HO-1 signaling pathway in liver

**FIGURE 5:**
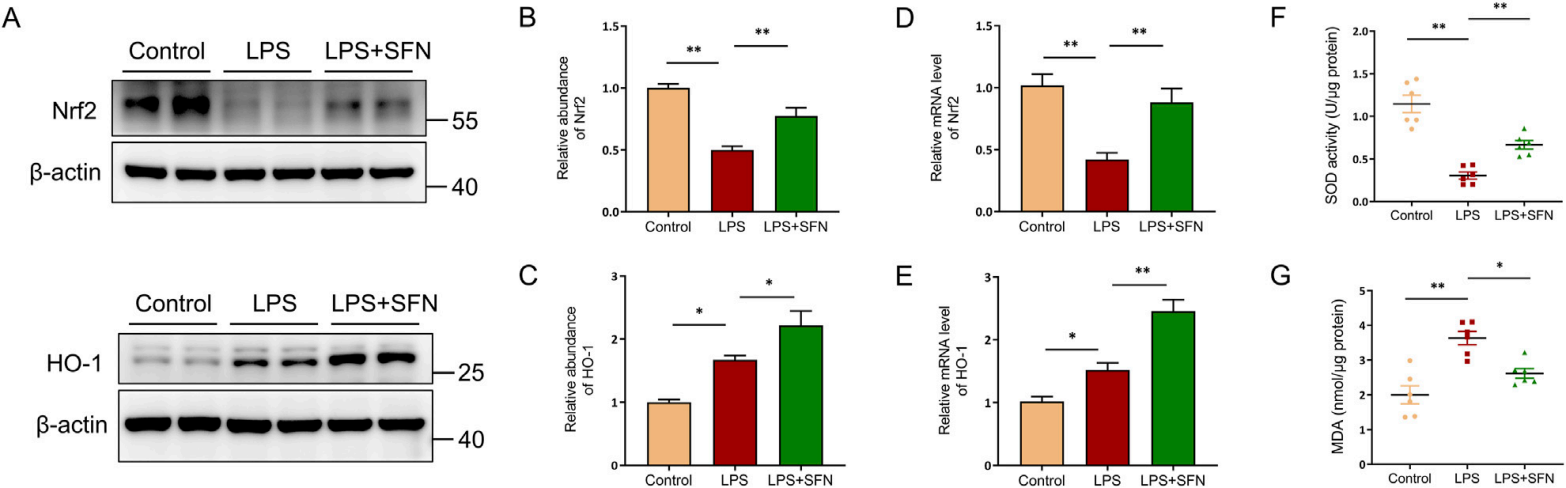
SFN activated Nrf2/HO-1 signaling pathway in liver. **(A–C)** Western blot analysis of Nrf2 and HO-1 in mice of control and LPS-treated mice with or without SFN pretreatment. **(A)**, and quantitative analysis **(B,C)**. **(D,E)** RT-qPCR analysis of Nrf2 and HO-1 in mice of control and LPS-treated mice with or without SFN pretreatment. **(F)** Measurement of SOD activity in the liver of different groups of mice. **(G)** Detection of MDA content in liver of different groups of mice. Data are presented as mean ± SEM of biologically independent samples. n = 6 per group. *P < 0.05, **P < 0.01. One-way ANOVA was used to analyze the data among multiple groups, followed by Tukey’s *post hoc* test.

To explore the underlying mechanism of SFN in hepatocyte pyroptosis, nuclear factor erythroid-2-related factor 2 (Nrf2) signaling was assessed. The abundance of Nrf2 and heme oxygenase-1 (HO-1) proteins was analyzed by Western blotting. Nrf2 and HO-1 protein expression, as well as the mRNA level of *Nfe2l2* and *Hmox1*, were significantly upregulated in SFN treatment mice compared with that in the LPS group (Figures 5A–E). Furthermore, compared to the LPS-treated mice, the superoxide dismutase (SOD) activity in the liver tissues of SFN-treated mice was significantly higher, while the hepatic level of malondialdehyde (MDA) was significantly lower (Figures 5F,G). These results suggest that SFN may suppress hepatocyte pyroptosis by reducing oxidative stress via the activation of the Nrf2/HO-1 signaling pathway.

## 4 Discussion

Sepsis causes ALI, a common clinical syndrome characterized by life-threatening organ dysfunction and is associated with high morbidity and mortality (Cecconi et al., 2018; Lelubre and Vincent, 2018). Progress in the development of therapeutic strategies for improving ALI outcomes and effective therapeutic strategies for the treatment of septic liver injury remain to be achieved. In the present study, we investigated the effect of SFN on the regulation of sepsis-induced acute liver injury, explored the effect of SFN on LPS-induced pyroptotic cell death, and provided evidence to mechanistically link Nrf2-HO-1 signaling with the regulation of SFN in hepatocyte pyroptosis and septic acute liver injury. Our study demonstrated that SFN ameliorated sepsis-exacerbated liver injury by reducing hepatocyte pyroptosis through Nrf2-HO-1 signaling, providing important insights into promising treatment approaches for ALI.

SFN is a natural isothiocyanate compound found in cruciferous vegetables, with robust antioxidant and anti-inflammatory characteristics (Yu et al., 2021; Shi et al., 2017; Russo et al., 2018). When compared to other well-studied phytochemicals like curcumin, SFN demonstrates superior oral bioavailability (Dinkova-Kostova and Kostov, 2012). Furthermore, unlike some synthetic Nrf2 activators that may cause constant hyperactivation of Nrf2, SFN, as a dietary compound, has an established safety profile (Cuadrado et al., 2019; Kensler et al., 2007). Its dual ability to potently activate the cytoprotective Nrf2 pathway while directly suppressing the pro-inflammatory pyroptotic cascade positions it as a uniquely attractive therapeutic agent. To explore the function of SFN in LPS-induced ALI, we pre-treated mice with SFN for 2 days before LPS injection, and the mice were sacrificed after 24 h LPS exposure. Plasma ALT and AST levels were measured to investigate the effect of SFN on ALI. The results showed that SFN significantly suppressed the upregulation of ALT and AST levels in the LPS-treated mice. Histological analysis of the liver confirmed the protective function of SFN, as reflected by smaller areas of necrosis. TUNEL staining showed that SFN reversed hepatocyte cell death, indicating that SFN ameliorated LPS-induced ALI. The overactivated inflammatory response is an important pathogenic factor in ALI; thus, we investigated the role of SFN in hepatic inflammation. Immunohistochemistry staining showed that F4/80^+^ macrophages and Ly6G^+^ neutrophils were increased in the liver of LPS-treated mice, whereas SFN decreased the infiltration of F4/80^+^ macrophages and Ly6G^+^ neutrophils. Similar trends in CXCL9, IL-6, MCP-1, and TNF-α mRNA levels were observed in the liver.

Pyroptosis is a type of programmed cell death that is closely correlated with the inflammatory response (Yu et al., 2021; Shi et al., 2017). RNA sequencing was performed to explore the effect of LPS on pyroptosis in the liver, and the results showed that hepatic pyroptotic cell death was induced in mice exposed to LPS. We explored the role of SFN in pyroptotic cell death by examining the expression of pyroptosis-related molecules. LPS-stimulated mRNA expression of NLRP3, Caspase1, IL-1β, and IL-18 was suppressed by SFN, as well as the protein abundance of GSDMD-N. SFN treatment reversed the increased plasma concentrations of IL-1β and IL-18 in LPS-exposed mice. These results indicated that SFN may inhibit LPS-induced pyroptotic cell death.

Oxidative stress is an important mechanism that contributes to cytotoxic effects when it exceeds the capacity of the cell to repair biomolecule oxidation (Sies, 2015). Excess reactive oxygen species (ROS), a leading cause of oxidative stress, is a significant factor that triggers pyroptosis by activating the NLRP3 inflammasome in response to pathological stimulus (Wang et al., 2024). Nrf2 is a major regulator of cytoprotective genes, with antioxidant and anti-inflammatory properties (Cuadrado et al., 2019). It is well established that the activation of Nrf2 provides effective protection from chronic inflammatory diseases and acute injury by upregulating downstream genes to suppress oxidative stress, such as HO-1 (O’Rourke et al., 2024; Wei et al., 2021; Wang et al., 2023). In this study, we investigated whether SFN could directly regulate Nrf2 signaling to modulate hepatocyte pyroptosis. In this study, we found that SFN blocked MDA levels in the livers of LPS-treated mice, while an adverse effect on SOD activity was observed. We verified the reduced hepatic Nrf2 expression in LPS-treated mice and demonstrated that SFN could reverse LPS-induced suppression of Nrf2. HO-1 is a vital antioxidant enzyme regulated by Nrf2 that blunts ROS generation and protects against cell death. Therefore, we explored the regulation of SFN on HO-1. As expected, SFN treatment induced HO-1 expression in liver. These data indicate that SFN specifically regulates Nrf2-HO-1 signaling in LPS-induced hepatocyte pyroptosis *in vivo*. Based on the above results, we propose that SFN ameliorates the pathological changes in ALI and uncovered a potential therapeutic strategy for LPS-induced ALI.

SFN’s therapeutic profile is distinguished from specific pyroptosis inhibitors and other hepatoprotective inhibitors by its multi-faceted mechanism. Unlike agents with narrower targets, SFN uniquely confers broad protection by concurrently inhibiting the pyroptosis executioner GSDMD and activating the antioxidant Nrf2 pathway, while also exhibiting favorable bioavailability. Furthermore, the dose of SFN (10 mg/kg/day) used in this study was selected based on its established efficacy in preclinical models of inflammation (Ran et al., 2023; Wu et al., 2016). Using the body surface area normalization method for dose translation, this murine dose equates to a human equivalent dose (HED) of approximately 0.81 mg/kg, or about 49 mg for a 60 kg adult (Reagan-Shaw et al., 2008). Notably, clinical trials have administered SFN at doses up to 300 µmol (equivalent to 54 mg) daily, indicating that our employed dose is within a pharmacologically relevant and clinically translatable range (Gasper et al., 2005; Zhang et al., 2020).

However, the present study still has several limitations. While the activation of the Nrf2-HO-1 pathway observed in this study strongly suggests it is the primary mechanism through which SFN exerts its protective effects, we acknowledge that the absence of genetic or pharmacological loss-of-function experiments represents a limitation. Future studies utilizing cell-specific -knockout models or specific inhibitors of Nrf2 and HO-1 will be essential to provide definitive proof of causality. Besides, the study lacked positive control group treated with pharmacological comparators which limited the interpretation of comparative efficacy of SFN on LPS-induced ALI. Subsequent research should include appropriate positive controls. Although LPS can induce severe liver injury within 24 h, it still has limitations to mimic clinically realistic acute liver injury caused by sepsis. To address this limitation, a sepsis model constructed with cecal ligation and puncture should be supplemented to explore the effects of SFN on liver injury in further study. Despite these limitations, our findings provide a valuable foundation for understanding the protective role of SFN and highlight the Nrf2-HO-1 pathway as a promising therapeutic target for sepsis-induced ALI.

Our preclinical study establishes that SFN ameliorates sepsis-induced organ injury by activating the Nrf2/HO-1 pathway to suppress pyroptosis, providing a strong mechanistic rationale for clinical translation. Future trials should validate this approach by monitoring key biomarkers—including liver enzymes) pyroptosis-driven cytokines, and metabolic indices such as HOMA-AD, with the ultimate goal of improving clinical outcomes like reduced liver failure incidence and enhanced survival in sepsis patients.

## 5 Conclusion

In our study, we discovered that SFN exerts an anti-pyroptotic property in ameliorating LPS-induced ALI, and that it is correlated with the activation of the Nrf2/HO-1 signaling pathway. In summary, the present study investigated the role and mechanism of SFN in the regulation of hepatocyte pyroptosis in septic ALI. SFN showed robust effects on reducing hepatocyte pyroptosis, hepatic inflammation, and liver injury. Furthermore, SFN restored Nrf2 expression and upregulated HO-1 expression, suggesting that SNF ameliorates septic liver injury by inhibiting hepatocyte pyroptosis via the Nrf2-HO-1 signaling pathway. Overall, SFN blunted the pathogenesis of LPS-induced ALI, implying that it is a potential natural product for the prevention and treatment of ALI.

## Data Availability

The datasets presented in this study can be found in online repositories. The names of the repository/repositories and accession number(s) can be found below: https://www.ncbi.nlm.nih.gov/geo/, GSE305948.
